# Partly Fermented Infant Formulae With Specific Oligosaccharides Support Adequate Infant Growth and Are Well-Tolerated

**DOI:** 10.1097/MPG.0000000000001360

**Published:** 2016-09-23

**Authors:** Frédéric Huet, Marieke Abrahamse-Berkeveld, Sebastian Tims, Umberto Simeoni, Gérard Beley, Christoph Savagner, Yvan Vandenplas, Jonathan O’B. Hourihane

**Affiliations:** ∗ Hôpital d’Enfants, Dijon, France; † Nutricia Research, Utrecht, The Netherlands; ‡ Center Hospitalier Universitaire Vaudois, Lausanne, Switzerland; § Maison de l’enfant Essey- lès- Nancy, Essey- lès- Nancy; || Center Hospitalier Universitaire d’Angers, Angers, France; ¶ Universitair Ziekenhuis Brussel, Vrije Universiteit Brussel, Brussels, Belgium; # Department of Paediatrics and Child Health, University College, Cork, Ireland.

**Keywords:** fermented formula, microbiota, safety, scGOS/lcFOS

## Abstract

**Objective:**

Fermented formulae (FERM) and a specific mixture of 90% short-chain galacto-oligosaccharides and 10% long-chain fructo-oligosaccharides (scGOS/lcFOS; 9:1) have a potential beneficial effect on gastrointestinal function and microbiota development in infants. The present study assessed the safety and tolerance of the combination of partly fermented infant milk formulae and scGOS/lcFOS compared with either 1 feature, in healthy term infants.

**Methods:**

Four hundred thirty-two infants were enrolled before 28 days of age and followed up to 17 weeks of age and assigned to 1 of the 4 groups: (i) formula with scGOS/lcFOS, (ii) scGOS/lcFOS + 15% FERM, (iii) scGOS/lcFOS + 50% FERM, or (iv) 50% fermented formula (50% FERM). Primary outcome was daily weight gain during intervention (equivalence criterion: difference in daily weight gain ≤3 g/day). Infants’ anthropometrics, formula intake, number, and type of (serious) AEs were monitored monthly. Stool samples were collected at baseline and after 17 weeks for analysis of physiological and microbiological parameters.

**Results:**

Equivalence of weight gain per day was demonstrated in both the intention-to-treat and per-protocol population, with a mean weight gain (SD) of 29.7 (6.1), 28.2 (4.8), 28.5 (5.0), and 28.7 (5.9) g/day for the groups i to iv respectively. No differences were observed in other growth parameters, formula intake, and the number or severity of AEs. In all scGOS/lcFOS-containing formulae, a beneficial effect of scGOS/lcFOS was observed, indicated by the lower pH, lower *Clostridium difficile* levels, and higher secretory immunoglobulin A levels.

**Conclusions:**

The partly fermented infant milk formulae containing the specific mixture scGOS/lcFOS were well-tolerated and resulted in normal growth in healthy infants.

**What Is Known**

- Historically, despite limited clinical evidence and knowledge on their mode of action, fermented formulae are used to promote digestibility and tolerance and are considered safe for use in infants (with respect to growth), although new studies are warranted.
- Infant formulae containing the specific prebiotic mixture of 90% short-chain galacto-oligosaccharides and 10% long-chain fructo-oligosaccharides (scGOS/lcFOS; 9:1) stimulate a closer to human milk gut microbiota, stool characteristics, have a beneficial effect on immune function and are considered safe for use in infants.
- To our knowledge, clinical evidence on the safety of infant formulae combining scGOS/lcFOS (9:1) and fermented formula is lacking.

**What Is New**

- Partly fermented infant formulae containing scGOS/lcFOS (9:1) can be considered as nutritionally adequate and safe for use in healthy, term infants.
- A beneficial effect of scGOS/lcFOS (9:1) on gut microbiota and stool characteristics was confirmed, irrespective of presence or dosage of fermented formula.

Exclusive human milk is the preferred feeding for all term newborn infants and provides a complete supply of nutrients to support growth and development in early life. In addition, human milk contains bioactive components that beneficially affect intestinal health, gut microbial colonization, and immune maturation (1–3). Because human milk feeding may not always be possible, human milk substitutes should provide nutritional and functional properties as close as possible to those of human milk.

Early nutrition plays an important role in the development and functioning of the gastrointestinal (GI) tract (4,5). Historically, fermented and/or acidified infant milk formulae have been used to promote digestibility and tolerance of formula. Only limited clinical evidence is, however, available to substantiate these suggested health benefits (6–10). Fermented formulae may reduce frequency and severity of gut discomfort (8), reduce the severity of infectious diarrhea, that is, fewer cases of dehydration or medical consultation (9) and stimulate the presence of intestinal bifidobacteria in healthy young infants (10). The ESPGHAN Committee on Nutrition, based on the limited evidence, cautiously reported that no safety concerns are expressed for the use of fermented formulae, although they emphasized more studies are warranted (7).

The development and maturation of the GI tract is accompanied by the dynamic enteric process of microbiota development, considered crucial for healthy infant development (11). Early nutrition has a strong effect on microbiota development: breast-fed infants typically have a microbiota dominated by bifidobacteria, whereas nonprebiotic supplemented formula-fed infants have a more diverse microbiota (12). Inspired by the molecular size distribution of the nondigestible oligosaccharides present in human milk (13), a specific mixture of 90% short-chain galacto-oligosaccharides and 10% long-chain fructo-oligosaccharides (scGOS/lcFOS; 9:1) was developed (14,15). Formulae containing this specific prebiotic mixture were shown to stimulate intestinal colonization with bifidobacteria resulting in beneficial effects on immune function (16–18). The addition of this prebiotic mixture prevents constipation, which is known to be more common in formula-fed than breast-fed infants (19). Safety and tolerance of formulae containing scGOS/lcFOS (9:1) has been demonstrated in several studies, with respect to growth (20–22) and digestive function (22–25). None of the studies included the prebiotic mixture in combination with fermented infant formula.

Supplementation of fermented formula with the specific prebiotic mixture scGOS/lcFOS could have a complementary, beneficial effect on the GI function of infants. First, the nutritional safety and adequacy of this new nutritional concept should be demonstrated following stringent evaluation (26–28).

In our study, safety was evaluated with daily weight gain as primary outcome parameter, and in addition, length gain, head circumference gain, and (serious) adverse events (AEs) were monitored. As an exploratory outcome, fecal physiological and microbial parameters were evaluated to confirm the beneficial effects of scGOS/lcFOS.

## SUBJECTS AND METHODS

### Subjects

Subjects were healthy infants recruited from mothers who could not or had chosen not to (continue to) breast-feed. Inclusion criteria were gestational age between 37 weeks and 42 weeks, birth weight between 2.5 and 4.5 kg, and postnatal age ≤28 days. Infants were excluded from the study if they had a congenital condition or illness that could interfere with the study or if they had a known or increased risk for cow's milk allergy. Other exclusion criteria were maternal gestational diabetes, participation in another clinical trial or investigator's uncertainty about the willingness or ability of the parents to comply with the protocol requirements.

The study was conducted according to ICH-GCP principles, and in compliance with the principles of the Declaration of Helsinki and with the local laws and regulations of the country where the study was performed. All participating centers obtained approval of their independent local Ethical Review Board.

Written informed consent was obtained from all parent(s)/guardian, ages 18 years or older, before enrolment to the study. This trial was registered in the Dutch Trial Register (NTR 2521).

### Participating Centers

The present study was conducted in a total of 24 study centers in France (n = 7; CHU of Dijon, American Memorial Hospital in Reims, CHU La conception in Marseille, CHU of Angers, CHU of Besancon, and a private pediatrician clinic in Essey les Nancy and in Floirac), Belgium (n = 10; UZ Brussel, Jessa Ziekenhuis Hasselt, private practice in Asse, Sainte Elisabeth Clinique in Namur, Heilig Hart Hospital in Roeleare, Aalsters Stedelijk Ziekenhuis in Aalst, Imelda Hospital in Bonheiden, GZA Sint Augustinus in Wilrijk, AZ Sint Blasius in Dendermonde, and AZ Sint Vicentius in Antwerp), and Ireland (n = 7; 2 tertiary referral hospitals: Cork University Hospital, National Maternity Hospital, Dublin, and 5 primary care centers: Medical Center and The Palms surgeries in Gorey, Meadowcroft Surgery in Wicklow, Slaney Medical Center in Enniscorthy, and Town Hall Surgery in Bray).

### Trial Design

The present study was a prospective, double-blind, controlled, randomized, parallel-group, multicenter controlled equivalence trial. Infants, whose parents intended to feed their infants formula, were assigned to 1 of the 4 groups, using a computerized random-number generator with sex and study site strata. Formulae were coded by the sponsor and both the investigators and the infant's parents were blinded to the formula. Baseline measurements were taken at enrolment and parents received the assigned infant formula with written preparation instructions, were advised to feed infants ad libitum, and were provided with recommendations for product intake volumes depending on the infant's weight.

During the intervention period infants were to be fed ad libitum exclusively with their allocated formula starting on the day of enrolment (0–28 days of age) until 17 weeks of age. The study consisted of a baseline visit and 4 subsequent hospital visits at 4, 8, 13, and 17 weeks of postnatal age. In addition, a follow-up phone call was performed 2 weeks after the last study visit. Parents were provided with diaries to record formula intake, GI symptoms, crying, sleeping, and stool characteristics every day during the week before the study visits. At each visit, investigators took anthropometric measures, reviewed compliance (based on evaluation of diaries), provided study product, and assessed the occurrence and outcome of (serious) AEs and use of concomitant medication and nutritional supplements by open questioning and/or diagnosis.

Furthermore, fecal samples were collected by the parents either during or shortly after the baseline visit, and just before the final visit at their infant's age of 17 weeks for analysis of the fecal physiological parameters and microbiological composition.

### Study Formulae

Each formula was powdered infant formula providing complete nutritional support for infants in the first 6 months of life. The formulae were isocaloric and contained per 100 mL a similar amount of 66 kcal energy, 1.35 g protein, 8.2–8.4 g carbohydrate, 3.0–3.1 g lipids, and vitamins and minerals according to Directive 2006/141/EC. The products varied only in the amount of formula produced by a specific fermentation process using *Bifidobacterium breve* and *Streptococcus thermophiles* (this process is called Lactofidus) and the presence of prebiotics, that is, 90% short-chain galacto-oligosaccharides and 10% long-chain fructo-oligosaccharides (0.8 g per 100 mL; Table 1). The fermentation process (Lactofidus) is followed by mild heat treatment. In total, 4 formulae were tested (i) a nonfermented commercially available formula with prebiotics (scGOS/lcFOS), (ii) a formula containing 15% fermented formula and prebiotics (scGOS/lcFOS + 15% fermented formulae [FERM]), (iii) a formula containing 50% fermented formula and prebiotics (scGOS/lcFOS + 50% FERM), and (iv) a commercially available formula containing 50% fermented formula without prebiotics (50% FERM). Hence, formula ii, iii, and iv are composed of a mixture of fermented and nonfermented infant formula in a ratio of 15:85 (ii) or 50:50 (iii, iv). The rationale to supplement prebiotics to a product with a level of 50% fermented formula was based on the previously observed beneficial effects on gut comfort (8). The level of 15% fermented formula was included to assess a potential dose dependency given the unknown effect on safety and tolerance of the newly developed formulae and has been shown to be well-tolerated (29). All formulae had a similar taste, smell, and color and were manufactured according to good manufacturing practices by Nutricia (Steenvoorde, France). The study products were to be stored at the sites at a secure and limited access storage area protected from extremes of light, temperature, and humidity.

**TABLE 1:** Composition of the intervention products (per 100 mL)

Per 100 mL	scGOS/lcFOS	scGOS/lcFOS +15% FERM	scGOS/lcFOS +50% FERM	50% FERM
scGOS/lcFOS 9:1, g	0.8	0.8	0.8	–
Fermented formula^*^	–	15%	50%	50%
Energy, kcal	66	66	66	66
Fat, g	3.0	3.0	3.0	3.1
Protein, g	1.35	1.35	1.35	1.36
Carbohydrates, g	8.2	8.2	8.2	8.4

15% FERM = infant formula consisting of 15% fermented infant formula; 50% FERM = infant formula consisting of 50% fermented infant formula; scGOS/lcFOS = short-chain galacto-oligosaccarides and long-chain fructo-oligosaccharides.

^*^Fermented by a specific combination of *Bifidobacterium breve* and *Streptococcus thermophilus*.

### Measurements

The primary outcome was weight gain per day from study entry to 17 weeks of age, that is, from ≤28 to 119 days of age. The secondary outcomes were length, head circumference, and mid-upper arm circumference. For safety assessment, the number, type, and severity of AEs were monitored and the use of medication. In addition, fecal physiological parameters and microbial composition were studied.

At each visit, the mean weight for each infant was registered by weighing them twice naked, on calibrated electronic scales, and in case of >100 g deviation an additional measurement was performed. The mean supine length of infants was registered by measuring them twice using a standard measuring board, and in case of >5 mm deviation an additional measurement was performed. A nonstretchable slotted insertion tape was used to measure head circumference in duplicate (or 3 times if deviation of >2 mm between measurements). The repeated measures in 1 infant were performed by the same investigator, if possible, using the same equipment. In case a third measurement was required, the 2 measures closest together were averaged as outcome measurement.

Diaries, filled in by the parents during the 7-day period before each visit, registered information on quantity of formula intake. In the feeding record, the number of feeds and the amount of formula prepared and left over per feed was registered. During the 17-week study period, (serious) AEs were documented by the investigators at each visit. Details recorded were start and stop date of the event, its severity, and actions that were taken. Moreover, the investigators documented (probability of) any relation with the study product.

### Fecal Parameters

Stool samples were collected by parents and brought to the study site for storage at or below –18°C for a maximum of 4 months. Thereafter, the samples were transported to Nutricia Research in insulated containers containing solid CO_2_ (dry ice) and stored at –80°C. Fecal parameters were determined for samples of visit 1 (baseline) and visit 5 (end of intervention period) for each study arm. Only samples of a subgroup of subjects were analyzed, which had a complete set of stool samples (both visits) with sufficient amount of stool for all analyses. In addition, samples from infant that used any systemic antibiotics any time after birth or that used thickeners added to formula during the study were excluded from analyses.

In the selected set of fecal samples the effect of the infant formulae was assessed on the following physiological and microbial parameters: pH, short-chain fatty acid (SCFA) levels (ie, acetate, propionate, butyrate, isobutyrate, valerate, and isovalerate), d- and l-lactate, secretory immunoglobulin A (SIgA), calprotectin, and presence of *Clostridium difficile*. The quantification methodology of these parameters have been described in more detail previously (25,30,31), except for calprotectin and presence of *C. difficile*. Human calprotectin levels in the stool samples were quantified with the Bühlmann Calprotectin ELISA kit (Bühlmann Laboratories AG, Schönenbuch, Switzerland) according to the manufacturer's protocol. The presence of *C. difficile* was detected with quantitative polymerase chain reaction utilizing primers 16S-Cldif -F (5′-GCA ACG CGA AGA ACC TTA CCT A-3′) and 16S-Cldif -R (5′-GAA GGG AAC TCT CCG ATT AAG GA-3′) in conjunction with probe 16S-Cldif (5′-VIC-TGA CAT CCC AAT GAC A-NFQ-MGB-3′), which is labeled with the reporter dye VIC and with the quencher NFQ-MGB (Applied Biosystems, Bleiswijk, The Netherlands). Genomic DNA of *C. difficile* LMG 21717 was used for the standard curve. The assay was performed with 1× TaqMan universal master mix (Applied Biosystems), 0.4 μmol/L of both primers, 0.2 μmol/L probe, and DNA extract in a final volume of 25 μL. The amplification program included an initial denaturation step at 95°C for 10 minutes, followed by 40 cycles of denaturation at 95°C for 15 seconds and primer annealing and extension at 60°C for 60 seconds.

### Statistics

During statistical analysis 3 comparisons of interest have been investigated. The effect of fermented formula is assessed by comparing scGOS/lcFOS + 15% FERM and scGOS/lcFOS + 50% FERM groups with the scGOS/lcFOS group. The effect of prebiotic addition is assessed by comparing the scGOS/lcFOS + 50% FERM group versus the 50% FERM group. The primary objective of the study was to test for equivalence of weight gain using the predefined margins of equivalence of 0.5 standard deviation (SD) of the difference in weight gain per day from randomization until 17 weeks of age. We set a minimum of 3 g/day (28) and a maximum value of 5 g/day for the equivalence margin to be used as clinically relevant margins in case 0.5 SD was exceeding these margins. To conclude equivalence, the 2-sided 90% confidence intervals for the differences in mean weight gain should lie entirely between –0.5 SD and +0.5 SD margins. This margin was also used to conclude equivalence for length gain and head circumference gain. The required sample size for 2 one-sided statistical testing using *α* = 0.05 and a power = 0.80 was 70 infants per intervention group. Allowing for a drop-out rate of 35%, a total of 432 infants (108 per group) had to be enrolled. The equivalence analysis was performed using parametric growth curves, this model describes the development of growth parameters (ie, weight) over time by a second-order polynomial curve correcting for sex, site was taken into account as a random effect, and each subject's intercept and slope were taken into account as random effects. Sensitivity analyses to evaluate robustness of results were performed using analysis of covariance on the 17 weeks of age measurement with the randomization measurement as covariate and a mixed model using the age variable as a categorical variable. In addition, to compare the intervention groups with the WHO Child Growth Standards of breastfed infants, an analysis of growth parameter *z* scores using WHO growth trajectories (32) were performed by using a mixed model with adjustment of baseline *z* score. In any other analyses, for continuous data 2 sample *t* tests were used and Wilcoxon rank sum tests (WR) were used in case of violation of normality assumption and/or presence of outliers. Categorical response parameters were analyzed by using Chi-square tests (C; Fisher exact tests [FEs] in case sparse cells occurred). The statistical analysis was performed by Nutricia Research using SAS (SAS Enterprise Guide 4.3 or higher) for Windows (SAS Institute Inc., Cary, NC). In the intention-to-treat (ITT) analysis, the data of all infants, except for those erroneously randomized were to be used. In the per-protocol (PP) analysis, eligibility of data was assessed on visit level, that is, a subject could be part of the PP population at baseline and visits 2 and 3, but excluded from the PP population at visits 4 and 5 if a protocol violation occurred after visit 3. In case of major protocol deviations, for example, if an inclusion criterion for birth weight was not met or lack of postbaseline visits, complete subjects were excluded from the PP dataset. Fecal composition data were evaluated using SigmaStat Statistical Analysis Software (Systat Software Inc., San Jose, CA). If <30% of the values were below limit of detection (BLD) or—in case of the quantitative polymerase chain reaction assays—not quantifiable (NQ), the value BLD was replaced by (detection limit/2) and the value NQ was replaced by (detection limit + limit of quantification)/2, after which WRs were performed. If >30% of the values were BLD or NQ, value BLD was replaced by “0” and the other values (including “NQ”) were replaced by “1,” after which *P* values were calculated using Chi-square test to assess differences in prevalence of the corresponding parameter.

## RESULTS

### Subject Characteristics

**FIGURE 1:**
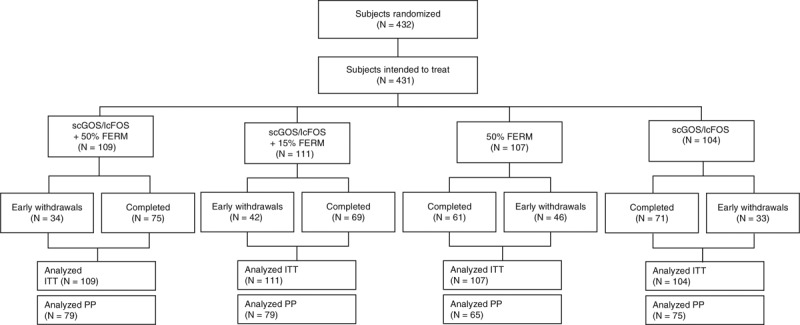
Disposition of study subjects per intervention group. ASR = all subjects randomized; FERM = fermented infant formula; ITT = intention-to-treat; PP = per protocol; scGOS/lcFOS = short-chain galacto-oligosaccarides and long-chain fructo-oligosaccharides.

From the total of 432 randomized subjects, included between October 2010 and September 2012, 16 subjects were excluded from the all-subjects-treated (AST) population because they did not consume any study product. One subject was diagnosed with hypothyroidism and was considered as not healthy, erroneously randomized, and excluded from ITT analysis (Fig. 1). A total of 276 subjects completed the study until 17 weeks of age, whereas 155 (36%) subjects dropped-out from the study prematurely. The number of subjects that completed the study was not apparently different between groups, although the number tended to be higher in the group combining scGOS/lcFOS with 50% FERM compared to that with 50% FERM only (FE *P* = 0.091). The most common reason for drop-out was an (serious) AE (n = 72), other reasons were withdrawal of informed consent (n=54), lost to follow-up (n = 15), protocol violation (n = 1), or other reasons (n = 13). There were no differences in reasons for drop-out between groups. At baseline, no differences in subject characteristics, including sex ratio, of the PP intervention groups were observed, except for a higher number of first-born infants in the scGOS/lcFOS with 50% FERM compared with the 50% FERM only group (FE *P* = 0.014; Table 2), which is considered to be a statistically significant difference by chance and without clinical relevance. Baseline anthropometric measures were not statistically different between treatment groups (Table 2).

**TABLE 2:** Demographics and baseline characteristics of infants per intervention group of the per protocol population (n = 298)

	Statistic	scGOS/lcFOS (N = 75)	scGOS/lcFOS +15% FERM (N = 79)	scGOS/lcFOS +50% FERM (N = 79)	50% FERM (N = 65)
Sex ^F^					
Male	n (%)	39 (52.0%)	37 (46.8%)	39 (49.4%)	33 (50.8%)
Female	n (%)	36 (48.0%)	42 (53.2%)	40 (50.6%)	32 (49.2%)
Mode of birth ^F^					
Natural	n (%)	61 (81.3%)	60 (75.9%)	58 (73.4%)	52 (80.0%)
C-section	n (%)	14 (18.7%)	19 (24.1%)	21 (26.6%)	13 (20.0%)
Ethnicity ^F^					
Caucasian/White	n (%)	67 (89.3%)	71 (89.9%)	72 (91.1%)	61 (93.8%)
Black	n (%)	2 (2.7%)	1 (1.3%)	3 (3.8%)	1 (1.5%)
Asian	n (%)	0 (0.0%)	1 (1.3%)	0 (0.0%)	0 (0.0%)
Combination	n (%)	3 (4.0%)	2 (2.5%)	3 (3.8%)	0 (0.0%)
Other	n (%)	3 (4.0%)	4 (5.1%)	1 (1.3%)	3 (4.6%)
Country ^F^					
Belgium	n (%)	28 (37.3%)	24 (30.4%)	25 (31.6%)	22 (33.8%)
France	n (%)	26 (34.7%)	34 (43.0%)	33 (41.8%)	25 (38.5%)
Ireland	n (%)	21 (28.0%)	21 (26.6%)	21 (26.6%)	18 (27.7%)
Order of birth ^F^					
First	n (%)	28 (37.3%)	29 (36.7%)	34 (43.0%)^*^	22 (33.8%)^*^
Second	n (%)	31 (41.3%)	34 (43.0%)	36 (45.6%)	27 (41.5%)
Third	n (%)	14 (18.7%)	12 (15.2%)	6 (7.6%)	16 (24.6%)
>Third	n (%)	2 (2.7%)	4 (5.1%)	3 (3.8%)	0 (0.0%)
Age at first visit, day ^W^	Median (min-max)	4 (0–28)	4 (0–28)	6 (0–28)	5 (0–28)
Gestational age, wk ^W^	Median (min-max)	39 (37–41)	40 (37–41)	40 (37–41)	39 (37–41)
Weight at birth, g ^T^	Mean ± SD	3336 ± 429	3378 ± 358	3370 ± 368	3444 ± 377
Length at birth, cm ^W^	Median (min-max)	50 (45–54)	50 (46–54)	50 (45–53)	50 (46–54)
Maternal age, yr ^T^	Mean ± SD	29.4 ± 4.9	30.3 ± 5.2	29.5 ± 5.1	29.5 ± 4.9
Maternal BMI, kg/m^2^ ^W^	Median (min-max)	24 (17–43)	24 (20–45)	25 (16–39)	25 (16–36)
Paternal age, yr ^T^	Mean ± SD	32.2 ± 5.4	32.6 ± 6.2	32.1 ± 5.6	32.7 ± 5.3
Paternal BMI, kg/m^2^ ^W^	Median (min-max)	25 (18–40)	26 (18–34)	26 (19–36)	26 (19–36)

BMI = body mass index; 15% FERM = infant formula consisting of 15% fermented infant formula; 50% FERM = infant formula consisting of 50% fermented infant formula; scGOS/lcFOS = short-chain galacto-oligosaccarides and long-chain fructo-oligosaccharides; SD = standard deviation.

^*^Values are significantly different. For the analysis of continuous data 2 sample *t* tests (T) were used and Wilcoxon rank sum tests (W) were used in case of violation of normality assumption and/or presence of outliers. Categorical response parameters were analyzed by using Fisher exact tests (F).

### Formula Consumption

The feeding history of subjects before the baseline visit, (any) breast-feeding and/or type of formula feeding, was not significantly different between groups in the PP population. In general, all study formulae were well accepted by the infants indicated by the high parental evaluation score (median score of 9 on evaluation scale 1–10 for all formulae; data not shown). The study product intake was not significantly different between subjects of each intervention group throughout the study (Table 3).

**TABLE 3:** Average daily product intake (mL/day) during the intervention period of the per protocol population (means ± standard deviation)

	scGOS/lcFOS (n = 75)	scGOS/lcFOS +15% FERM (n = 79)	scGOS/lcFOS +50% FERM (n = 79)	50% FERM (n = 65)
Visit 2 (n)	699 ± 124 (67)	685 ± 131 (72)	697 ± 111 (69)	728 ± 129 (60)
Visit 3 (n)	772 ± 109 (65)	768 ± 140 (66)	782 ± 161 (70)	825 ± 155 (56)
Visit 4 (n)	827 ± 151 (59)	817 ± 139 (65)	830 ± 129 (67)	867 ± 159 (54)
Visit 5 (n)	852 ± 123 (55)	859 ± 128 (62)	878 ± 137 (62)	928 ± 159 (52)

Diaries were included in the analysis if product intake was recorded for at least 3 days. The data were analyzed using a *t* test for each comparison of interest. 15% FERM = infant formula consisting of 15% fermented infant formula; 50% FERM = infant formula consisting of 50% fermented infant formula; scGOS/lcFOS = short-chain galacto-oligosaccarides and long-chain fructo-oligosaccharides.

### Infant Growth Patterns

**FIGURE 2:**
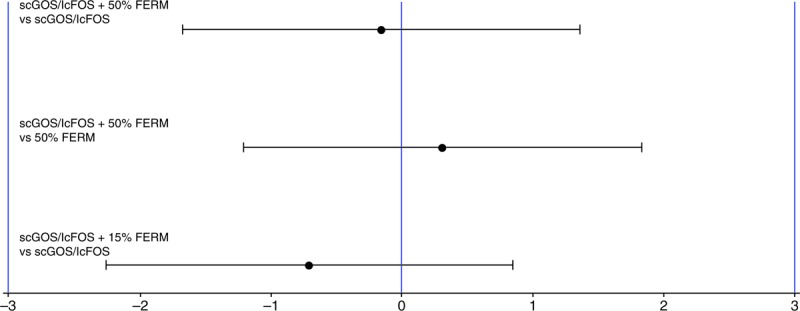
Graphical display of the difference in means of the daily weight gain (g/d) equivalence analysis for the intervention group comparisons of interest. The equivalence analysis was performed using parametric growth curves correcting for sex, site was taken into account as random effect, and each subject's intercept and slope taken into account as random effects. FERM = fermented infant formula; scGOS/lcFOS = short-chain galacto-oligosaccarides and long-chain fructo-oligosaccharides.

**FIGURE 3:**
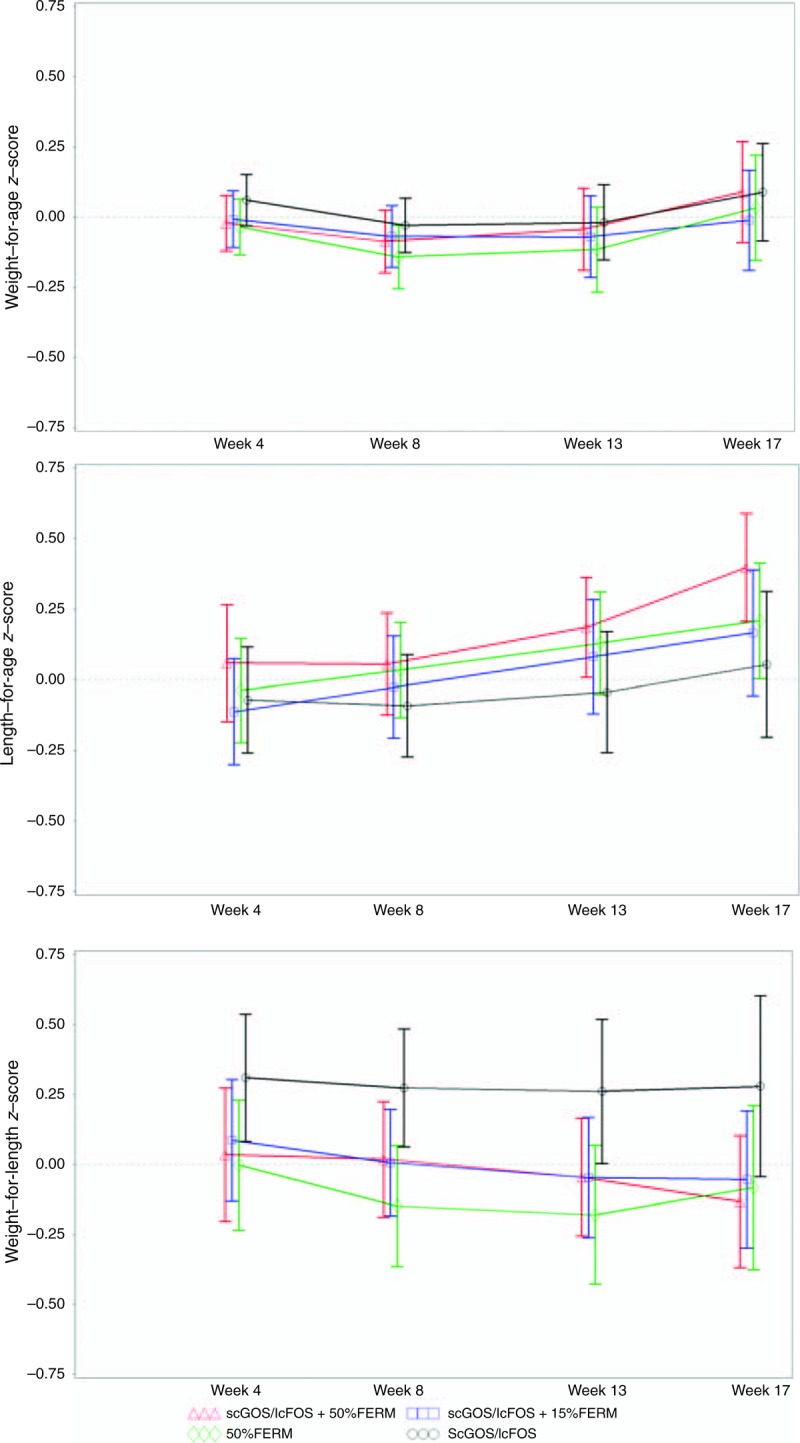
Weight-for-age (upper), length-for-age (middle), and weight-for-length (lower) WHO *z* score: plot of predicted value plus 95% confidence interval (CI) from mixed model analysis of z-score for the PP population per intervention group. FERM = fermented infant formula; scGOS/lcFOS = short-chain galacto-oligosaccarides and long-chain fructo-oligosaccharides.

In all comparisons of interest, the 2-sided 90% confidence interval (CI) of the mean lay well within the predefined equivalent margins (Fig. 2), demonstrating equivalence in weight gain for the PP population. Equivalence of weight gain per day for the same comparison groups was also confirmed for the ITT population (data not shown). Length gain, head circumference gain, and mid-upper arm circumference gain during the study period were not different between intervention groups in these 3 comparisons of interest (Table 4). Moreover, in line with weight gain per day, equivalence in length and head circumference gain per day during the intervention period was demonstrated for these parameters in all but 1 comparison of interest. Equivalence could not be demonstrated for the length gain of scGOS/lcFOS versus scGOS/lcFOS + 50% FERM groups in the PP population, with an apparently higher gain in length in the latter group. In comparison with the WHO Growth Standards based on growth of exclusively breastfed infants, the mean *z* score values (including their 95% CI) for weight-for-age, length-for-age, and weight-for-length in all intervention groups lay within or close to the + 0.5 to –0.5 range (Fig. 3).

**TABLE 4:** Average gain in infant growth, length, and head circumference (mean ± standard deviation) during the intervention period (baseline to 17 weeks) in the per protocol population

	scGOS/lcFOS (n = 75)	scGOS/lcFOS +15% FERM (n = 79)	scGOS/lcFOS +50% FERM (n = 79)	50% FERM (n = 65)
Weight gain in g/day (n)	29.734 ± 6.121 (57)	28.538 ± 5.064 (65)	28.727 ± 5.903 (64)	28.235 ± 4.833 (52)
Length gain in mm/day (n)	1.078 ± 0.195 (56)	1.078 ± 0.180 (64)	1.135 ± 0.127 (63)	1.092 ± 0.147 (52)
Head circumference gain in mm/day (n)	0.572 ± 0.128 (47)	0.559 ± 0.122 (54)	0.566 ± 0.098 (52)	0.580 ± 0.083 (42)

15% FERM = infant formula consisting of 15% fermented infant formula; 50% FERM = infant formula consisting of 50% fermented infant formula; scGOS/lcFOS = short-chain galacto-oligosaccarides and long-chain fructo-oligosaccharides.

### (Serious) Adverse Events

The safety analysis was performed on the all-subjects-treated population. In total, 28 serious AEs (SAE) were reported for 27 subjects (6.5%) during the entire study period. The prevalence of SAEs was apparently higher in the scGOS/lcFOS group, but was only significantly different from the scGOS/lcFOS + 15% FERM group (13.3% vs 2.7%, respectively; FE *P* = 0.007; Table 5). An initial higher than expected rate of pyelonephritis was recorded in the scGOS/lcFOS group. Assessing the medical records in more detail revealed inconsistencies for the use of this medical term; 1 case was found to be an urinary tract infection and 1 case a direct result of a congenital anatomical anomaly causing vesicoureteral reflux, reducing the incidence to the level of normal background incidence. Only 3 SAEs were reported to be possibly (n = 2; reflux in scGOS/lcFOS + 50% FERM group and abdominal pain in 50% FERM group) or probably (n = 1) related to the study products, the latter being a possible case of cow's milk allergy in the scGOS/lcFOS + 50% FERM intervention group. The rest of the SAEs were “unlikely to be related” or “not related” to the intake of the intervention products. Overall, there was no safety concern. No differences were observed in the frequency and type of AEs. The most commonly observed (37%) were GI disorders (n = 154), consisting mainly of abdominal pain, gastroesophageal reflux and vomiting, and respiratory system disorders (n = 110; 26%), of which rhinitis and upper respiratory tract infection were the most common.

**TABLE 5:** Number and percentage of subjects with at least 1 serious adverse event presented by preferred term in the all subjects treated population

n (%)	scGOS/lcFOS (n = 98)	scGOS/lcFOS +15% FERM (n = 110)	scGOS/lcFOS +50% FERM (n = 106)	50% FERM (n = 102)
Allergy			1 (0.9%)	
Fever	1 (1.0%)		1 (0.9%)	1 (1.0%)
Pyloric stenosis	1 (1.0%)		1 (0.9%)	
Abdominal pain				1 (1.0%)
Gastroesophageal reflux			1 (0.9%)	1 (1.0%)
Vomiting	2 (2.0%)			
Fracture	1 (1.0%)			
Urinary tract infection		1 (0.9%)	1 (1.0%)	
Apnoea	1 (1.0%)			
Bronchitis		1 (0.9%)	1 (0.9%)	
Pneumonia		1 (0.9%)	1 (0.9%)	1 (1.0%)
Upper respiratory tract infection	2 (2.0%)			
Surgery	1 (1.0%)			
Varicella	1 (1.0%)			
Pyelonephritis	3 (3.1%)^*^			
Total	13 (13.3%)^†^	3 (2.7%)^†^	7 (6.6%)	4 (3.9%)

15% FERM = infant formula consisting of 15% fermented infant formula; 50% FERM = infant formula consisting of 50% fermented infant formula; scGOS/lcFOS = short-chain galacto-oligosaccarides and long-chain fructo-oligosaccharides.

^*^Although officially recorded as 3 cases of pyelonephritis, detailed assessment of the medical records revealed an inconsistency for the use of this medical term, 1 case was a urinary tract infection and 1 case a direct result of a congenital anatomical anomaly causing vesicoureteral reflux.

^†^Values are significantly different, based on Fisher exact test (difference in number of subjects between groups).

### Fecal Physiological and Microbial Parameters

After the intervention, that is, at 17 weeks of age, the measured fecal physiological and microbial parameters of the infants from the scGOS/lcFOS only arm confirmed its beneficial effect (33), that is, lower pH, high relative amounts of acetate, low relative levels of the other measured SCFAs, high occurrence of measurable d- and l-lactate, high levels of SIgA and low occurrence of *C difficile*, a taxonomic group known to be overrepresented in formula-fed infants as compared to breast-fed infants (34) (Table 6). No differences in fecal parameters were observed comparing both scGOS/lcFOS + 15% FERM and scGOS/lcFOS + 50% FERM to the scGOS/lcFOS only group, except for a higher pH in the scGOS/lcFOS + 50% FERM group (WR *P* = 0.028; Table 6). In contrast, the fecal parameters of the infants from the 50% FERM only arm were always significantly different compared with the scGOS/lcFOS + 50% FERM group, with the exception of the SCFAs acetate, propionate, and a tendency for higher butyrate levels in the 50% FERM group (C *P* = 0.066; Table 6). The levels of fecal calprotectin are in line with previously reported data from formula-fed infants and no differences between groups were observed in the comparisons of interest (Table 6; (35)).

**TABLE 6:** Physiological parameters and presence of *Clostridium difficile* in fecal samples at 17 weeks of age

Parameter		Statistic shown	scGOS/lcFOS (N = 30)	scGOS/lcFOS +15% FERM (N = 30)	scGOS/lcFOS +50% FERM (N = 30)	50% FERM (N = 30)
Acetate ^W^ (mmol/kg wet weight faeces)		Median (min-max)	81.9 (65.7–93.7)	78.6 (63.4–103.2)	74.5 (59.0–93.2)	62.9 (50.3–74.2)
Propionate ^W^ (mmol/kg wet weight faeces)		Median (min-max)	8.0 (2.9–14.6)	8.1 (4.5–16.0)	15.3 (5.5–18.0)	14.3 (6.4–20.1)
Butyrate ^C^ (mmol/kg wet weight faeces)		Median (min-max)	0.9 (0.0–3.1)	0.8 (0.0–3.0)	1.2 (0.0–3.0)	5.3 (1.1–8.6)
Isobutyrate ^C^	0	n (%)	23 (79.3%)	23 (76.7%)	17 (58.6%)^*^	7 (23.3%)^*^
	1	n (%)	6 (20.7%)	7 (23.3%)	12 (41.4%)^*^	23 (76.7%)^*^
Valerate ^C^	0	n (%)	27 (93.1%)	25 (83.3%)	29 (100.0%)^*^	26 (86.7%)^*^
	1	n (%)	2 (6.9%)	5 (16.7%)	0 (0.0%)^*^	4 (13.3%)^*^
Isovalerate ^C^	0	n (%)	16 (55.2%)	14 (46.7%)	13 (44.8%)^*^	1 (3.3%)^*^
	1	n (%)	13 (44.8%)	16 (53.3%)	16 (55.2%)^*^	29 (96.7%)^*^
l-lactate ^C^	0	n (%)	4 (13.8%)	5 (16.7%)	8 (26.7%)^*^	21 (70.0%)^*^
	1	n (%)	25 (86.2%)	25 (83.3%)	22 (73.3%)^*^	9 (30.0%)^*^
d-lactate ^C^	0	n (%)	9 (31.0%)	14 (46.7%)	13 (43.3%)^*^	22 (73.3%)^*^
	1	n (%)	20 (69.0%)	16 (53.3%)	17 (56.7%)^*^	8 (26.7%)^*^
*Costridium difficile* ^C^	0	n (%)	22 (73.3%)	24 (80.0%)	24 (80.0%)^*^	13 (43.3%)^*^
	1	n (%)	8 (26.7%)	6 (20.0%)	6 (20.0%)^*^	17 (56.7%)^*^
SIgA (μg/g wet weight feces) ^**W**^		Median (min-max)	1245.9 (144.7–3006.1)	1371.5 (57.0–4093.3)	1424.4^*^ (84.9–3346.4)	499.7^*^ (45.6–5145.4)
Calprotectin (μg/g wet weight feces) ^**W**^		Median (min-max)	225.8 (48.6 – 1815.3)	240.0 (3.1–1377.2)	181.0 (8.6–4584.9)	207.1 (44.3–1926.3)
pH ^**W**^		Median (min-max)	5.6^†^ (5.0–7.3)	5.7 (5.1–7.6)	6.1^*^^,^^†^ (5.2–7.6)	6.9^*^ (5.6–8.2)

^W^For the analysis a Wilcoxon rank sum tests (W) was used due to violation of normality assumption and/or presence of outliers. ^C^Categorical response parameters (0 = absent, 1 = present) were analyzed by using Chi-square tests (C). 15% FERM = infant formula consisting of 15% fermented infant formula; 50% FERM = infant formula consisting of 50% fermented infant formula; scGOS/lcFOS = short-chain galacto-oligosaccarides and long-chain fructo-oligosaccharides; SIgA = secretory immunoglobulin A.

^*^Significantly different values between groups for the tested comparison of interest: scGOS/lcFOS + 50% FERM versus 50% FERM only.

^†^Significantly different values between groups for the tested comparison of interest: scGOS/lcFOS + 15% FERM or scGOS/lcFOS + 50% FERM versus scGOS/lcFOS only.

## DISCUSSION

To our knowledge, this is the first study evaluating the safety of a partly fermented infant formula supplemented with prebiotics. The primary outcome of the study is the demonstrated equivalence in infant weight gain per day during the intervention period analyzed according to both Intention to Treat and Per Protocol, fulfilling the safety criteria of the American Academy of Pediatrics (28). No differences in number and type of (serious) AEs were observed. The obtained anthropometric measures in all intervention groups were close to the WHO Growth Standards (mean *z* scores within ±0.5 SD), indicating that the formulae combining the specific prebiotic mixture (scGOS/lcFOS 9:1) with fermented infant formula support an adequate infant growth.

The current findings in the 50% FERM group confirm the previously assessed adequate infant growth and tolerance based on the long history of use (6) and based on the secondary anthropometric outcomes of several studies which were smaller-scaled (10), using a thickened fermented formula (8), or performed in older (9) or preterm infants (36). Likewise, the outcomes of the present study for the scGOS/lcFOS only intervention group confirm previous findings indicating a normal infant growth (20–22). A meta-analysis demonstrated a slightly increased weight gain (weight gain increase of 0.97 g/day, 95% CI 0.24–1.70) but not length or head circumference gain, in infants consuming a prebiotic-containing formula (37). In the present study, we confirmed a small increase (0.31 g/day) in mean weight gain per day during the intervention period when prebiotics were added to a partly fermented formula.

All formulae were well accepted by parents and the presence of the prebiotics and/or fermented formula did not affect formula consumption. Only 1 SAE was found to be probably related to study product intake, being an allergic reaction to cow's milk experienced in the scGOS/lcFOS + 50% FERM group evidently relating to the presence of dairy protein rather than fermented formula or prebiotics. The same intervention group contained 1 case of symptomatic gastroesophageal reflux indicated by the investigators to be possibly related to the study product intake, as did the 50% FERM intervention group. The overall prevalence of symptomatic gastroesophageal reflux was, however, low and also similar between intervention groups. The percentage of infants with at least 1 SAE was similar in all intervention groups with partly fermented formula, but slightly higher in the scGOS/lcFOS only intervention group, reaching statistical significance compared with the scGOS/lcFOS + 15%FERM intervention group. The observed number of SAE in the scGOS/lcFOS group is, however, within the normal range for an infant population and quite similar to the values reported in comparable studies for (prebiotic) formula- or breast-fed infants (38,39). Moreover, none of the SAEs in the scGOS/lcFOS intervention group was indicated to be related to the product and some rather the result of chance, for example, 4 of the 13 SAEs were infants experiencing varicella, fracture, surgery, and pyloric stenosis. Overall, the reported types of (serious) AEs are typical for young infants and no difference in the frequency per type of (serious) AEs was observed between the different formula groups. Moreover, the drop-out rate and reasons for dropping out were similar between intervention groups. Hence, there are no safety concerns for use of products combining prebiotics and fermented formula in healthy, term infants.

The presence of scGOS/lcFOS (9:1) in nonfermented infant formula was found to modulate the microbiota of infants toward a more “breast-fed like” composition with more bifidobacteria and less pathogenic bacteria such as clostridia-related species (16,18,20,31). This observed modulation in microbiota was found to be associated with differences in metabolic activity of the microbiota resulting in a reduction in stool pH, an SCFA pattern containing a higher proportion of acetate, a lower proportion of propionate or other SCFAs, and increased levels of fecal SIgA (16,20,31). The present study confirms this established bifidogenic effect of scGOS/lcFOS (9:1) when added to partly fermented formulae, indicated by the lower percentage of *C difficile*, the higher occurrence of measurable lactate levels, the decreased pH, and increased SIgA levels reported for the scGOS/lcFOS + 50% FERM group compared with the 50% FERM only group. Moreover, no differences in fecal parameters were demonstrated between the formulae containing scGOS/lcFOS (9:1), irrespective of the presence or dosage of fermented formula (scGOS/lcFOS + 15% FERM and scGOS/lcFOS + 50% FERM vs scGOS/lcFOS only). Hence, the present study reconfirms the beneficial effect of scGOS/lcFOS, which was not affected by the presence of fermented formula.

In conclusion, a partly fermented formula containing a specific mixture of prebiotics supports adequate growth in early life firstly based on the weight gain equivalence between intervention groups in the ITT and PP populations and, secondly, on the similarity in growth development compared to the WHO growth standards. In addition, these formulae were well-tolerated and safe based on growth and AE outcomes. As a next step, the potential effect of partly fermented formulae with scGOS/lcFOS on GI function is under investigation.

## References

[1] Field CJ The immunological components of human milk and their effect on immune development in infants. *J Nutr* 2005; 135:1–4. 15623823 10.1093/jn/135.1.1

[2] Garcia C Duan RD Brévaut-Malaty V Bioactive compounds in human milk and intestinal health and maturity in preterm newborn: an overview. *Cell Mol Biol (Noisy-le-grand)* 2013; 59:108–131. 25326648

[3] Jost T Lacroix C Braegger C Impact of human milk bacteria and oligosaccharides on neonatal gut microbiota establishment and gut health. *Nutr Rev* 2015; 73:426–437. 26081453 10.1093/nutrit/nuu016

[4] Thompson AL Monteagudo-Mera A Cadenas MB Milk- and solid-feeding practices and daycare attendance are associated with differences in bacterial diversity, predominant communities, and metabolic and immune function of the infant gut microbiome. *Front Cell Infect Microbiol* 2015; 5:3. 25705611 10.3389/fcimb.2015.00003 PMC4318912

[5] Reisinger KW de Vaan L Kramer BW Breast-feeding improves gut maturation compared with formula feeding in preterm babies. *J Pediatr Gastroenterol Nutr* 2014; 59:720–724. 25111221 10.1097/MPG.0000000000000523

[6] Van de Heijning BJ Berton A Bouritius H GI symptoms in infants are a potential target for fermented infant milk formulae: a review. *Nutrients* 2014; 6:3942–3967. 25255831 10.3390/nu6093942 PMC4179197

[7] Agostoni C Goulet O Kolacek S Fermented infant formulae without live bacteria. *J Pediatr Gastroenterol Nutr* 2007; 44:392–397. 17325568 10.1097/01.mpg.0000258887.93866.69

[8] Roy P Aubert-Jacquin C Avart C Benefits of a thickened infant formula with lactase activity in the management of benign digestive disorders in newborns. *Arch Pediatr* 2004; 11:1546–1554. 15596352 10.1016/j.arcped.2004.10.001

[9] Thibault H Aubert-Jacquin C Goulet O Effects of long-term consumption of a fermented infant formula (with *Bifidobacterium breve* c50 and *Streptococcus thermophilus* 065) on acute diarrhea in healthy infants. *J Pediatr Gastroenterol Nutr* 2004; 39:147–152. 15269618 10.1097/00005176-200408000-00004

[10] Mullie C Yazourh A Thibault H Increased poliovirus-specific intestinal antibody response coincides with promotion of *Bifidobacterium longum*-infantis and *Bifidobacterium breve* in infants: a randomized, double-blind, placebo-controlled trial. *Pediatr Res* 2004; 56:791–795. 15347767 10.1203/01.PDR.0000141955.47550.A0

[11] Wopereis H Oozeer R Knipping K The first thousand days—intestinal microbiology of early life: establishing a symbiosis. *Pediatr Allergy Immunol* 2014; 25:428–438. 24899389 10.1111/pai.12232

[12] Bäckhed F Roswall J Peng Y Dynamics and stabilization of the human gut microbiome during the first year of life. *Cell Host Microbe* 2015; 17:690–703. 25974306 10.1016/j.chom.2015.04.004

[13] Boehm G Stahl B Oligosaccharides from milk. *J Nutr* 2007; 137 (3 suppl 2):847S–849S. 17311985 10.1093/jn/137.3.847S

[14] Stahl B Thurl S Zeng J Oligosaccharides from human milk as revealed by matrix-assisted laser desorption/ionization mass spectrometry. *Anal Biochem* 1994; 223:218–226. 7887467 10.1006/abio.1994.1577

[15] Boehm G Fanaro S Jelinek J Prebiotic concept for infant nutrition. *Acta Paediatr Suppl* 2003; 91:64–67. 14599044 10.1111/j.1651-2227.2003.tb00648.x

[16] Knol J Scholtens P Kafka C Colon microflora in infants fed formula with galacto- and fructo-oligosaccharides: more like breast-fed infants. *J Pediatr Gastroenterol Nutr* 2005; 40:36–42. 15625424 10.1097/00005176-200501000-00007

[17] Arslanoglu S Moro GE Schmitt J Early dietary intervention with a mixture of prebiotic oligosaccharides reduces the incidence of allergic manifestations and infections during the first two years of life. *J Nutr* 2008; 138:1091–1095. 18492839 10.1093/jn/138.6.1091

[18] Moro G Arslanoglu S Stahl B A mixture of prebiotic oligosaccharides reduces the incidence of atopic dermatitis during the first six months of age. *Arch Dis Child* 2006; 91:814–819. 16873437 10.1136/adc.2006.098251 PMC2066015

[19] Scholtens PA Goossens DA Staiano A Stool characteristics of infants receiving short-chain galacto-oligosaccharides and long-chain fructo-oligosaccharides: a review. *World J Gastroenterol* 2014; 20:13446–13452. 25309075 10.3748/wjg.v20.i37.13446 PMC4188896

[20] Costalos C Kapiki A Apostolou M The effect of a prebiotic supplemented formula on growth and stool microbiology of term infants. *Early Hum Dev* 2008; 84:45–49. 17433577 10.1016/j.earlhumdev.2007.03.001

[21] Schmelzle H Wirth S Skopnik H Randomized double-blind study of the nutritional efficacy and bifidogenicity of a new infant formula containing partially hydrolyzed protein, a high beta-palmitic acid level, and nondigestible oligosaccharides. *J Pediatr Gastroenterol Nutr* 2003; 36:343–351. 12604972 10.1097/00005176-200303000-00008

[22] Decsi T Arató A Balogh M Randomised placebo controlled double blind study on the effect of prebiotic oligosaccharides on intestinal flora in healthy infants. *Orv Hetil* 2005; 146:2445–2450. 16408384

[23] Magne F Hachelaf W Suau A Effects on faecal microbiota of dietary and acidic oligosaccharides in children during partial formula feeding. *J Pediatr Gastroenterol Nutr* 2008; 46:580–588. 18493215 10.1097/MPG.0b013e318164d920

[24] Boehm G Lidestri M Casetta P Supplementation of a bovine milk formula with an oligosaccharide mixture increases counts of faecal bifidobacteria in preterm infants. *Arch Dis Child Fetal Neonatal Ed* 2002; 86:F178–F181. 11978748 10.1136/fn.86.3.F178 PMC1721408

[25] Bakker-Zierikzee AM Alles MS Knol J Effects of infant formula containing a mixture of galacto- and fructo-oligosaccharides or viable *Bifidobacterium animalis* on the intestinal microflora during the first 4 months of life. *Br J Nutr* 2005; 94:783–790. 16277782 10.1079/bjn20051451

[26] Koletzko B Shamir R Ashwell M Quality and safety aspects of infant nutrition. *Ann Nutr Metab* 2012; 60:179–184. 22699763 10.1159/000338803

[27] Hernell O Current safety standards in infant nutrition—a European perspective. *Ann Nutr Metab* 2012; 60:188–191. 22699765 10.1159/000338210

[28] American Academy of Pediatrics, Taskforce on clinical testing of infant formulas. Clinical testing of infant formulas with respect to nutritional suitability for term infants. (Report prepared under FDA contract 223-86-2117), 1988 *http://www.fda.gov/Food/GuidanceRegulation/GuidanceDocumentsRegulatoryInformation/InfantFormula/ucm170649.htm*. Accessed August 2, 2016.

[29] Garcette K Bellaïche M Serena Survey: changes in minor functional symptoms and in the quality of life of infants receiving Bledilait 1. *Med Enfance* 2007; 27:3–7.

[30] Knol J Boehm G Lidestri M Increase of faecal bifidobacteria due to dietary oligosaccharides induces a reduction of clinically relevant pathogen germs in the faeces of formula-fed preterm infants. *Acta Paediatr Suppl* 2005; 94:31–33. 16214763 10.1111/j.1651-2227.2005.tb02152.x

[31] Scholtens PA Alliet P Raes M Fecal secretory immunoglobulin A is increased in healthy infants who receive a formula with short-chain galacto-oligosaccharides and long-chain fructo-oligosaccharides. *J Nutr* 2008; 138:1141–1147. 18492847 10.1093/jn/138.6.1141

[32] WHO Multicentre Growth Reference Study Group. WHO Child growth standards based on length/height, weight and age. *Acta Paediatr* 2006; 450:76–85. 10.1111/j.1651-2227.2006.tb02378.x 16817681

[33] Oozeer R van Limpt K Ludwig T Intestinal microbiology in early life: specific prebiotics can have similar functionalities as human-milk oligosaccharides. *Am J Clin Nutr* 2013; 98:561S–571S. 23824728 10.3945/ajcn.112.038893

[34] Azad MB Konya T Maughan H Gut microbiota of healthy Canadian infants: profiles by mode of delivery and infant diet at 4 months. *CMAJ* 2013; 185:385–394. 23401405 10.1503/cmaj.121189 PMC3602254

[35] Savino F Castagno E Calabrese R High faecal calprotectin levels in healthy, exclusively breast-fed infants. *Neonatology* 2010; 97:299–304. 19887860 10.1159/000255161

[36] Campeotto F Suau A Kapel N A fermented formula in pre-term infants: clinical tolerance, gut microbiota, down-regulation of faecal calprotectin and up-regulation of faecal secretory IgA. *Br J Nutr* 2011; 105:1843–1851. 21426607 10.1017/S0007114510005702

[37] Mugambi MN Musekiwa A Lombard M Synbiotics, probiotics or prebiotics in infant formula for full term infants: a systematic review. *Nutr J* 2012; 11:81. 23035863 10.1186/1475-2891-11-81 PMC3544682

[38] Chouraqui JP Grathwohl D Labaune JM Assessment of the safety, tolerance, and protective effect against diarrhea of infant formulas containing mixtures of probiotics or probiotics and prebiotics in a randomized controlled trial. *Am J Clin Nutr* 2008; 87:1365–1373. 18469260 10.1093/ajcn/87.5.1365

[39] Puccio G Cajozzo C Meli F Clinical evaluation of a new starter formula for infants containing live *Bifidobacterium longum* BL999 and prebiotics. *Nutrition* 2007; 23:1–8. 17189085 10.1016/j.nut.2006.09.007

